# Astaxanthin, a xanthophyll carotenoid, prevents development of dextran sulphate sodium-induced murine colitis

**DOI:** 10.3164/jcbn.18-47

**Published:** 2018-08-11

**Authors:** Shigeki Sakai, Atsushi Nishida, Masashi Ohno, Osamu Inatomi, Shigeki Bamba, Mitsushige Sugimoto, Masahiro Kawahara, Akira Andoh

**Affiliations:** 1 Department of Medicine, Shiga University of Medical Science, Seta-Tsukinowa, Otsu 520-2192, Japan

**Keywords:** inflammatory bowel disease, reactive oxygen species, carotenoid

## Abstract

Astaxanthin is a xanthophyll carotenoid, which possesses strong scavenging effect on reactive oxygen species. In this study, we examined the effect of astaxanthin on dextran sulfate sodium (DSS)-induced colitis in mice. Experimental colitis was induced by the oral administration of 4% w/v DSS in tap water in C57BL/6J mice. Astaxanthin was mixed with a normal rodent diet (0.02 or 0.04%). Astaxanthin significantly ameliorated DSS-induced body weight loss and reduced the disease activity index. The ameliorating effects was observed in a dose-dependent manner. Immunochemical analyses showed that astaxanthin markedly suppressed DSS-induced histological inflammatory changes (inflammatory cell infiltration, edematous changes and goblet cell depletion). Plasma levels of malondialdehyde and 8-hydroxy-2-deoxyguanosine were significantly reduced by the administration of 0.04% astaxanthin. Astaxanthin significantly suppressed the mucosal mRNA expression of IL-1β, IL-6, TNF-α, IL-36α and IL-36γ. Astaxanthin blocked the DSS-induced translocation of NF-κB p65 and AP-1 (c-Jun) into the nucleus of mucosal epithelial cells, and also suppressed DSS-induced mucosal activation of MAPKs (ERK1/2, p38 and JNK). In conclusion, astaxanthin prevented the development of DSS-induced colitis via the direct suppression of NF-κB, AP-1 and MAPK activation. These findings suggest that astaxanthin is a novel candidate as a therapeutic option for the treatment of inflammatory bowel disease.

## Introduction

Inflammatory bowel disease (IBD) is a term for two disorders [Crohn’s disease (CD) and ulcerative colitis (UC)] that are characterized by chronic inflammation of the gastrointestinal (GI) tract. IBDs are relapsing and remitting conditions that afflict millions of people throughout the world. The pathophysiology of IBD remains unclear, but previous studies have demonstrated that the excess mucosal immune response against dietary factors and the gut microbiota has a crucial role.^(1–3)^

Oxygen metabolism produces reactive oxygen species (ROS). ROS are products of normal aerobic metabolism, but they are excessively generated under pathophysiological conditions.^(4)^ Oxidative stress induced by excessive ROS generation causes uncontrolled interactions between ROS and neighboring components such as proteins, lipids, nucleic acids and carbohydrates, and leads to an imbalance of the redox homeostasis which is responsible for a variety of abnormalities associated with chronic disorders.^(5)^ For example, increased production of ROS in the vascular endothelial cells induces the appearance of abnormalities associated with atherosclerosis and cardiovascular diseases.^(6)^ The lipid accumulation in a steatotic liver has been reported to be associated with higher generation of ROS resulting in mitochondrial dysfunction, subsequently leading to hepatocyte apoptosis or necrosis.^(7)^ In the GI tract, oxidative stress leads to damages of the mucosal barrier functions and induces bacterial invasion, which in turn stimulates the immune and inflammatory response reported in IBD.^(8)^ In murine colitis models, the absence of macrophages or neutrophils leads to a decrease in ROS generation, reduces mucosal expression of proinflammatory cytokines, and ameliorates intestinal inflammation and damage.^(9)^

As exogenous antioxidants, carotenoids such as α- and β-carotene and lutein play important roles in preventing oxidative damage by excessive ROS generation through their scavenging activities. They can penetrate the cellular bilayer membrane due to their lipophilic nature and perform their antioxidative actions in different regions of the human body. Astaxanthin is a xanthophyll carotenoid, which can be produced by a variety of seaweeds and microorganisms.^(10)^ Astaxanthin has a strong scavenging effect on ROS and other prooxidant molecules. Astaxanthin exerts 10-fold greater antioxidative activity than other carotenoids including lutein, canthaxanthin and β-carotene through its unique molecular structure characterized by polar ionic rings and non-polar conjugated double carbon bonds.^(5)^ Astaxanthin also blocks peroxidation at the lipid membrane level with a greater efficacy than other known antioxidant compounds.^(5)^ Furthermore, astaxanthin has been reported to prevent activation of proinflammatory transcription factor NF-κB as a result of H_2_O_2_ neutralization.^(5)^ The antioxidant properties and beneficial effects of astaxanthin have been previously reported in various pathological conditions, such as atherosclerosis and cardiovascular disease,^(6)^ cerebrovascular disease,^(11)^ steatotic liver disease,^(7)^ ultraviolet-induced skin damage,^(12)^ and others.^(13,14)^ However, the effects of astaxanthin on an IBD model or IBD patients have still not been widely investigated despite an understanding of the role of oxidative stress in the inflammatory response in the gut.

This study examined how astaxanthin modulates the development of dextran sulfate sodium (DSS)-induced colitis and identifies a potential therapeutic role for astaxanthin in patients with IBD.

## Materials and Methods

### Animals and DSS colitis

C57BL/6J mice (six to eight week-old females; CLEA Japan Inc., Tokyo, Japan) were housed under specific pathogen-free conditions. Astaxanthin was provided by AstaReal Co., Ltd. (Tokyo, Japan). Astaxanthin was mixed with the powder form of a normal rodent diet (0.02 or 0.04%). The administration of astaxanthin was started 7 days before DSS administration. Mice were treated with the oral administration of 4% w/v DSS (molecular weight 5,000; Wako Pure Chemical Industries, Osaka, Japan) in tap water. The volume of water intake was measured daily to determine the amount of DSS consumed per mouse. We divided mice into 6 groups; control group (Control), astaxanthin 0.02% group (AX 0.02), astaxanthin 0.04% group (AX 0.04), DSS group (DSS), DSS plus 0.02% astaxanthin group (DSS+AX 0.02), DSS plus 0.04% astaxanthin group (DSS+AX 0.04). Mice were sacrificed on day 10, and histological and biochemical analyses were performed. This study was approved by the Research Center for Animal Life Science and Use Committee at the Shiga University of Medical Science (Otsu, Japan) (Permit number: 2015-1-5).

### Histological evaluation of DSS-induced colitis

The disease activity index (DAI) described previously,^(15)^ was used for evaluation of mucosal inflammation. Formalin-fixed colon sections were stained with Hematoxylin-Eosin (HE). Histologic evaluations were performed in a blinded fashion using a validated scoring system.^(16)^

### Human colonic epithelial cell line (HT-29)

The human colon epithelial cell line, HT-29, was purchased from the American Type Culture Collection (ATCC, Manassas, VA). The cells were maintained according to the instructions of ATCC.

### Measurement of plasma malondialdehyde (MDA) and 8-oxo-2'deoxyguanosine (8-OHdG)

Plasma MDA levels were measured using an ELISA kit (OxiSelect TBARS Assay Kit) purchased from CELL BIOLABS (San Diego, CA). Plasma 8-OHdG levels were measured using an ELISA kit purchased from the Japan Institute for Control of Aging (Fukuroi, Shizuoka, Japan).

### Immunohistochemical staining for NF-κB p65, phosphorylated (P)-c-Jun

Immunohistochemical analysis of tissue samples was performed according to the method described previously.^(17)^ The used antibodies are listed in Supplemental Table 1*.

### Real-time PCR analysis

The TRIzol reagent (Invitrogen, Carlsbad, CA) was used for extraction of total RNA. Total RNA was converted to cDNA using Superscript II (Invitrogen). The real-time PCR was performed using the Light Cycler 480 system (Roche Applied Science, Tokyo, Japan) and the reagent SYBR Premix Ex Taq II (TAKARA, Otsu, Japan). The data were normalized to β-actin for each target molecule, and were expressed as fold-increases relative to the data of the medium alone (no stimulation). The oligonucleotide primers are listed in Supplemental Table 2*.

### Nuclear and cytoplasmic proteins

The nuclear proteins from tissue were obtained using a CelLytic NuCLEAR Extraction Kit (Sigma-Aldrich Co., St Louis, MO). Nuclear proteins were subjected to immunoblot for NF-κB p65 and c-Jun. The cytoplasmic proteins were obtained by lysing buffer (20 mM Tris-HCl, 2 mM EDTA, 2 mM EGTA, 150 mM NaCl, 400 mM sodium fluoride, 4 mM sodium orthovanadate, 1% NP-40 Nonidet, containing cOmplete mini). Cytoplasmic proteins were subjected to immunoblot for MAPKs. The enhanced chemiluminescence immunoblot system (GE Healthcare, Little Chalfont, UK) was used for signal detection. The used antibodies are listed in Supplemental Table 2*.

The HT-29 cells were stimulated with TNF-α (100 ng/ml) in the presence or absence of astaxanthin for 10 min, and then the nuclear and cytoplasmic proteins were extracted.

### Immunoblot analysis

Ten µg of protein from each sample was subjected to SDS-PAGE on a 4–20% gradient gel under reducing conditions. Proteins were then transferred onto a nitrocellulose membrane. The detection was performed using an enhanced chemiluminescence Western blotting system (Amersham). The used antibody is listed in Supplemental Table 1*.

### Statistical analysis

Data are presented as means ± SEM. One-way ANOVA with Bonferroni post hoc tests were performed for statistical analysis. *P* values <0.05 were considered significant.

## Results

**Fig. 1:**
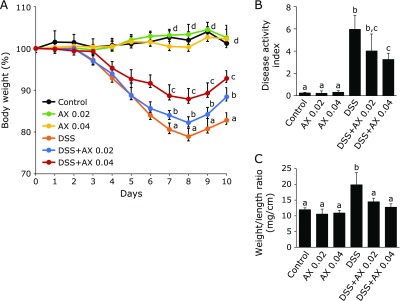
The effect of astaxanthin on the development of DSS colitis. Astaxanthin was mixed with a normal rodent diet (0.02 or 0.04%). (A) Body weight, (B) disease activity index and (C) colonic weight/length. All data are means ± SEM (*n* = 5/group). Values not sharing a letter denote significant differences (*p*<0.05). AX, astaxanthin.

To evaluate the effects of astaxanthin on DSS-colitis, mice were treated with astaxanthin for 7 days prior to the start of DSS administration. Astaxanthin had no effects on body weight of control mice (Fig. 1A). On the other hand, body weight was significantly reduced in the DSS group as compared with control mice, and administration of astaxanthin significantly ameliorated DSS-induced body weight loss. The ameliorating effects of 0.04% astaxanthin on body weight loss were significantly stronger than those of 0.02% astaxanthin. As shown in Fig. 1B, the DAI was significantly higher in the DSS group than in the control mice, and the administration of astaxanthin significantly reduced the DAI of the DSS group. Colon weight/length ratio, a marker of tissue edema, was significantly higher in the DSS group than in the control group (Fig. 1C). The administration of astaxanthin significantly reduced the DSS-induced elevation of colon weight/length ratio.

**Fig. 2:**
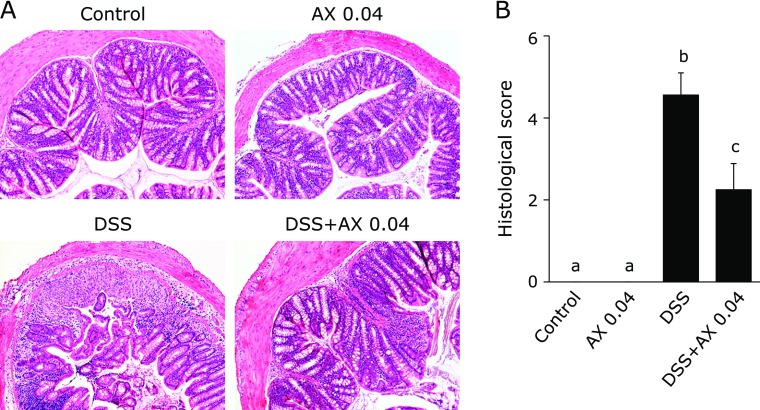
Histological evaluation of DSS-colitis. (A) Histological picture of the colonic tissue on day 10 (original magnification ×100). (B) Histological score. All data are means ± SEM (*n* = 5/group). Values not sharing a letter denote significant differences (*p*<0.05). AX, astaxanthin.

Histological pictures are shown in Fig. 2A. The administration of astaxanthin showed no histological changes. DSS treatment induced a marked infiltration of inflammatory cells in the mucosa, edematous changes of the mucosa, depletion of goblet cells and disruption of epithelial cells, and astaxanthin markedly suppressed these inflammatory changes induced by DSS administration. The histological inflammatory score was significantly lower in the DSS plus 0.04% astaxanthin group than in the DSS mice (Fig. 2B). Thus, oral administration of astaxanthin prevented the induction of DSS colitis.

**Fig. 3:**
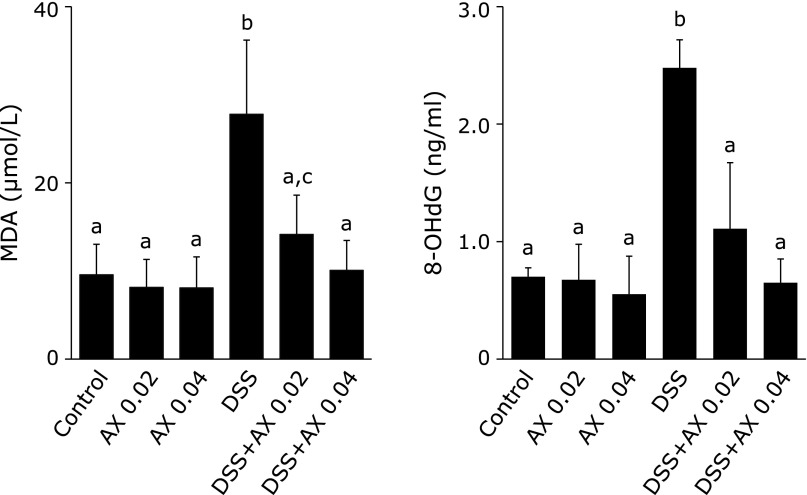
The effect of astaxanthin on plasma index of lipid peroxidation (MDA) and oxidative DNA damage (8-OHdG). Astaxanthin significantly reduced the elevation of plasma MDA and 8-OHdG levels. All data are means ± SEM (*n* = 5/group). Values not sharing a letter denote significant differences (*p*<0.05). AX, astaxanthin.

The anti-oxidative activities of astaxanthin were evaluated by plasma levels of malondialdehyde (MDA) and 8-hydroxy-2-deoxyguanosine (8-OHdG). MDA is a widely used index of lipid peroxidation^(18)^ and 8-OHdG is a marker of oxidative DNA damage.^(19)^ As shown in Fig. 3, plasma MDA and 8-OHdG levels were significantly higher in the DSS group than in the control group, and elevated MDA and 8-OHdG levels were significantly reduced in the DSS plus 0.04% astaxanthin group. These findings indicate that DSS-induced oxidative stress was significantly reduced by the addition of astaxanthin.

**Fig. 4:**
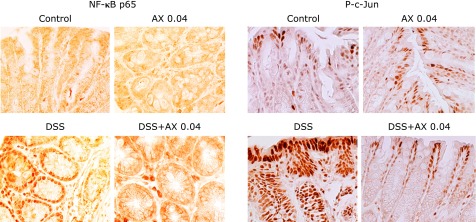
The effect of astaxanthin on NF-κB and AP-1 activation in DSS mice. Immunohistochemical staining was performed for detection of mucosal activation of NF-κB p65 and c-Jun (original magnification ×400). NF-κB p65 and P-c-Jun was detected in the nucleus of the colonic epithelial cells in DSS-mice, but this was markedly blocked in the DSS plus 0.04% astaxanthin group. AX, astaxanthin.

**Fig. 5:**
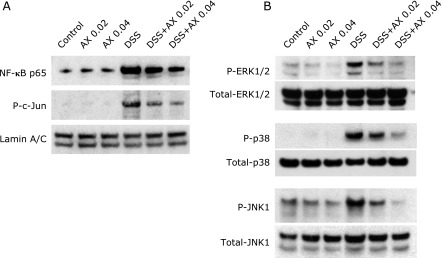
Immunoblot analyses for evaluation of NF-κB, AP-1 and MAPKs activation in colonic epithelial cells isolated from DSS mice. (A) The nuclear protein was extracted from epithelial cells of DSS mice, and NF-κB and phosphorylated (P) c-Jun were evaluated by immunoblotting. (B) The cytoplasmic protein was extracted from epithelial cells of DSS mice, and total and phosphorylated ERK1/2, p38 and JNK1 were evaluated by immunoblotting. The picture is representative of three independent experiments. AX, astaxanthin.

NF-κB and AP-1 (c-Jun) are major transcriptional factors regulating the expression of a number of inflammatory genes.^(20,21)^ Activation of NF-κB and AP-1 (c-Jun) was evaluated by immunohistochemical staining in the tissues. As shown in Fig. 4, DSS treatment induced a translocation of NF-κB p65 into the nucleus of epithelial cells, but this response was markedly suppressed in the DSS and 0.04% astaxanthin group. Activation of AP-1 was analyzed using anti-phosphorylated (P) c-Jun antibodies. AP-1 (P-c-Jun) was detected in the nucleus of the epithelial cells of the DSS group, but this was markedly blocked in the DSS plus 0.04% astaxanthin group. This phenomenon of NF-κB and AP-1 was also investigated by immunoblotting. Nuclear protein was extracted from colonic epithelial cells of the mice, and immunoblotting was performed using anti-NF-κB p65 and P-c-Jun antibodies. DSS treatment increased translocation of NF-κB p65 and P-c-Jun into the nucleus (Fig. 5A), and these responses were suppressed in the DSS plus astaxanthin group (Fig. 5A). These findings indicate that astaxanthin suppresses the DSS-induced activation of NF-κB and AP-1 in the colonic epithelial cells.

It has previously been reported that mitogen-activated protein kinase (MAPKs) mediate various inflammatory responses in the GI tract.^(22,23)^ To assess how astaxanthin modulates MAPK activation, we investigated the effects of astaxanthin on MAPK activation in the colonic epithelial cells isolated from DSS-mice. Astaxanthin alone had no effects on MAPK activation in colon tissue. Administration of DSS induced a marked phosphorylation of MAPKs including ERK1/2, p38 and JNK, and administration of astaxanthin suppressed DSS-induced MAPK activations in a dose-dependent manner (Fig. 5B).

**Fig. 6:**
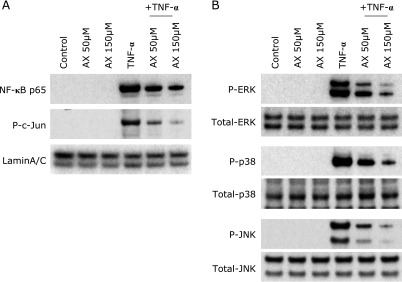
Immunoblot analyses for evaluation of NF-κB, AP-1 and MAPK activation in HT-29 colonic epithelial cells. HT-29 cells were stimulated with TNF-α (100 ng/ml) in the presence or absence of astaxanthin for 10 min. Then, the nuclear and cytoplasmic proteins were extracted. (A) Accumulation of NF-κB p65 and phosphorylated (P) c-Jun in the nucleus were evaluated by immunoblotting. (B) The cytoplasmic proteins were subjected to immunoblotting to evaluate phosphorylated ERK1/2, p38 and JNK1. The picture is representative of three independent experiments. AX, astaxanthin.

To investigate the direct effect of astaxanthin on colonic epithelial cells, we used colonic epithelial cell line HT-29 cells. As shown in Fig. 6, immunoblot analysis using nuclear proteins indicated that stimulation with TNF-α (100 ng/ml) induced a rapid translocation of NF-κB p65 and c-Jun into the nucleus, and astaxanthin markedly suppressed these responses. Similarly, TNF-α (100 ng/ml) induced a rapid phosphorylation of MAPKs (ERK1/2, p38 and JNK), and these responses were suppressed by the addition of astaxanthin. These observations indicate direct anti-inflammatory effects of astaxanthin on intestinal epithelial cells.

**Fig. 7:**
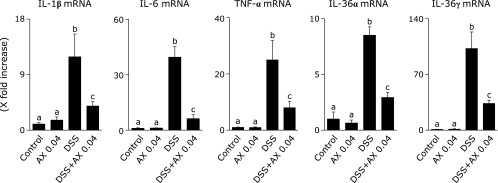
The effect of astaxanthin on the mRNA expression of proinflammatory cytokines. Mucosal mRNA expression for TNF-α, IL-1β, IL-6, IL-36α and IL-36γ was evaluated by real time-PCR. The mRNA expressions of cytokines were converted to a value relative to β-actin mRNA expression and revealed as a relative increase to the results for control mice. All data are means ± SEM (*n* = 5/group). Values not sharing a letter denote significant differences (*p*<0.05). AX, astaxanthin.

Various cellular and molecular mechanisms are involved in the development of DSS-colitis. Among them, proinflammatory cytokines have been reported to play a major role.^(24)^ We evaluated mucosal expression of the mRNAs for proinflammatory cytokines, such as IL-1β, IL-6, TNF-α, IL-36α and IL-36γ. As shown in Fig. 7, the mRNA expression of these cytokines was significantly elevated in the DSS group compared to the control group, but the addition of astaxanthin significantly reduced the elevated mRNA expression of proinflammatory cytokines.

## Discussion

In this study, to assess the therapeutic potential of astaxanthin in human IBD, its effects on the development of DSS murine colitis was investigated. Astaxanthin induced improvements of various factors in DSS colitis, including: clinical and histological parameters, mRNA expression of inflammatory cytokines (IL-1β, IL-6, TNF-α, IL-36α and IL-36γ), activation of transcription factors NF-κB and AP-1 (c-Jun), and MAPK phosphorylation. These were accompanied by a reduction in plasma oxidative stress markers, MDA and 8-OHdG. To our knowledge, this is the first study to propose a consideration that astaxanthin, a natural antioxidant product of seaweed and microorganisms, is a candidate for the treatment of IBD.

Oxygen metabolism, which is required for mammalian cell survival, produces ROS. Under physiological conditions, a certain level of ROS is eliminated by endogenous antioxidant capacity, and this maintains intestinal homeostasis. However, an excessive oxidant load induced by elevated ROS generation can irresistibly reinforce membrane permeability, alter the inflammatory response, and lead to lipid and protein modifications, DNA damage, apoptosis, and carcinogenesis.^(25)^ This condition is referred to as oxidative stress and is observed in many human diseases, such as cancer, diabetes, cardiovascular diseases, atherosclerosis, and chronic obstructive pulmonary disease.^(26)^

While the precise mechanisms underlying the etiology of IBD have not been fully defined, previous studies have suggested that oxidative stress as well as multiple factors including genetic and environmental factors contribute to the development of IBD.^(8)^ Among the many enzymes involved in ROS generation, mucosal NADPH oxidase has been reported to play an important role in the pathogenesis of IBD through induction of an imbalance in redox homeostasis.^(27)^ Xanthine oxidase plays a main role in ROS generation in the intestinal mucosa, leading to GI tract injuries,^(28)^ while myeloperoxidase is active in the inflamed mucosa of UC patients and contributes to the progression of malignancies.^(29)^ These factors suggest that oxidative stress may be a therapeutic target for IBD and that agents which eliminate oxidative stress may have a therapeutic potential.

NF-κB and AP-1 are critical transcription factors which mediate transcriptional activation of many inflammatory genes^(17,30)^ and are aberrantly activated in IBD.^(31)^ NF-κB can modulate the permeability of the intestinal epithelial cell (IEC) layer and is associated with IEC homeostasis.^(32)^ Previous studies have shown that oxidative stress stimulates NF-κB signaling and facilitates the mucosal inflammatory response in IBD.^(6,29)^ Under the normal physiological condition, NF-κB and AP-1 locate in the cytoplasm, and inflammatory stimuli induce translocation of the NF-κB and AP-1 molecules into the nucleus and activate transcription of inflammatory genes. In this study, immunohistochemical analysis showed that blockade of DSS-colitis by astaxanthin was accompanied by suppression of nuclear accumulation of transcription factor NF-κB and AP-1 (c-Jun). Immunoblot analysis also showed that the translocation of NF-κB p65 and AP-1 (c-Jun) into the nucleus of colonic epithelial cells was markedly suppressed in the astaxanthin plus DSS group. Furthermore, *in vitro* experiments using HT-29 cells suggested a direct inhibitory effect of astaxanthin on activation of NF-κB and AP-1 in colonic epithelial cells. Thus, astaxanthin ameliorated mucosal inflammation via the suppression of NF-κB and AP-1 activation.

The MAPK (ERK, p38 and JNK) pathway is a critical downstream signaling pathway of ROS stimulation, and its activation has been implicated in the pathogenesis of IBD. Several studies have revealed that the MAPK pathways contribute to various biological responses, such as inflammation, cell growth and differentiation, cell death and survival, in multiple cell types.^(33)^ Phosphorylated MAPKs can bind to and activate the target kinases, translocate into the nucleus and activate transcription of pro-inflammatory genes. In this study, we confirmed that astaxanthin dose-dependently suppressed DSS-induced phosphorylation of MAPKs in colonic epithelial cells isolated from DSS-mice.

We demonstrated that astaxanthin suppressed the mucosal expression of mRNAs for proinflammatory cytokines (IL-1β, IL-6, TNF-α and IL-36).The increased expression of IL-1β, IL-6, TNF-α and IL-36 has been reported to play a key role in the pathogenesis of human IBD and experimental colitis.^(34,35)^ The expression of IL-1, IL-6, TNF-α and IL-36 has been reported to be mediated by intracellular signal transduction involving the NF-κB pathway and the activation of MAPKs.^(35,36)^ Thus, according to the results in this study, it can be suggested that astaxanthin might exert anti-inflammatory activity through the inhibition of the NF-κB p65/MAPK signaling pathway followed by reduced expression of proinflammtory cytokines.

Complementary and alternative medicine (CAM) means the medical products and practices that are not part of standard medical treatments and used together with or instead of conventional medicine.^(37)^ CAM is sometimes required by the patients who are feeling an insufficient response to standard medical treatments or having a concern over side effects of drugs. There is an increasing number of reports concerning clinical application of nonconventional therapeutic approaches with antioxidant effects for IBD.^(8)^ These include ROS generation inhibitors, hormones, functional dietary interventions, and natural or synthetic substances that inhibit antioxidant enzymes.^(8)^ These antioxidant therapies may have fewer side effects, lower costs, and better treatment responses, offering new hope to IBD patients. The strong inhibitory effects of astaxanthin on the development of DSS colitis suggest a clinical application of astaxanthin for IBD patients as a CAM. Astaxanthin may be useful for induction and maintenance therapy in mild to moderately active UC patients who failed to achieve sufficient response to optimized mesalamine and/or who feel concerns to use immune modulators or biologics. Astaxanthin may also be useful as an adjuvant therapy for remission maintenance on optimized mesalamine. The clinical usefulness of astaxanthin in IBD patients has not been previously reported, but our findings strongly suggest that further clinical studies are warranted.

In conclusion, astaxanthin prevented the development of DSS-induced colitis through the direct suppression of NF-κB, AP-1 and MAPK activation. This was accompanied by a suppression of mucosal cytokine expression. These findings suggest that astaxanthin is a candidate as a therapeutic option for the treatment of IBD.

## References

[1] Sheehan D, Moran C, Shanahan F (2015). The microbiota in inflammatory bowel disease. J Gastroenterol.

[2] Sun M, Wu W, Liu Z, Cong Y (2017). Microbiota metabolite short chain fatty acids, GPCR, and inflammatory bowel diseases. J Gastroenterol.

[3] Podolsky DK (2002). Inflammatory bowel disease. N Engl J Med.

[4] Lü JM, Lin PH, Yao Q, Chen C (2010). Chemical and molecular mechanisms of antioxidants: experimental approaches and model systems. J Cell Mol Med.

[5] Galasso C, Corinaldesi C, Sansone C (2017). Carotenoids from marine organisms: biological functions and industrial applications.. Antioxidants (Basel)..

[6] Mangge H, Becker K, Fuchs D, Gostner JM (2014). Antioxidants, inflammation and cardiovascular disease. World J Cardiol.

[7] Li S, Takahara T, Fujino M (2017). Astaxanthin prevents ischemia-reperfusion injury of the steatotic liver in mice. PLoS One.

[8] Tian T, Wang Z, Zhang J (2017). Pathomechanisms of oxidative stress in inflammatory bowel disease and potential antioxidant therapies. Oxid Med Cell Longev.

[9] Muthupalani S, Ge Z, Feng Y (2012). Systemic macrophage depletion inhibits *Helicobacter bilis*-induced proinflammatory cytokine-mediated typhlocolitis and impairs bacterial colonization dynamics in a BALB/c *Rag2*^−/−^ mouse model of inflammatory bowel disease. Infect Immun.

[10] Ambati RR, Phang SM, Ravi S, Aswathanarayana RG (2014). Astaxanthin: sources, extraction, stability, biological activities and its commercial applications--a review. Mar Drugs.

[11] Bahonar A, Saadatnia M, Khorvash F, Maracy M, Khosravi A (2017). Carotenoids as potential antioxidant agents in stroke prevention: a systematic review. Int J Prev Med.

[12] Tominaga K, Hongo N, Fujishita M, Takahashi Y, Adachi Y (2017). Protective effects of astaxanthin on skin deterioration. J Clin Biochem Nutr.

[13] Kuraji M, Matsuno T, Satoh T (2016). Astaxanthin affects oxidative stress and hyposalivation in aging mice. J Clin Biochem Nutr.

[14] Okazaki Y, Okada S, Toyokuni S (2017). Astaxanthin ameliorates ferric nitrilotriacetate-induced renal oxidative injury in rats. J Clin Biochem Nutr.

[15] Berberat PO, A-Rahim YI, Yamashita K (2005). Heme oxygenase-1-generated biliverdin ameliorates experimental murine colitis. Inflamm Bowel Dis.

[16] Obermeier F, Kojouharoff G, Hans W, Schölmerich J, Gross V, Falk W (1999). Interferon-gamma (IFN-gamma)-and tumour necrosis factor (TNF)-induced nitric oxide as toxic effector molecule in chronic dextran sulphate sodium (DSS)-induced colitis in mice. Clin Exp Immunol.

[17] Tak PP, Firestein GS (2001). NF-κB: a key role in inflammatory diseases. J Clin Invest.

[18] Nahar Z, Sarwar MS, Safiqul Islam (2013). Determination of serum antioxidant vitamins, glutathione and MDA levels in panic disorder patients. Drug Res (Stuttg)..

[19] Alak G, Yeltekin AC, Tas IH (2017). Investigation of 8-OHdG, CYP1A, HSP70 and transcriptional analyses of antioxidant defence system in liver tissues of rainbow trout exposed to eprinomectin. Fish Shellfish Immunol.

[20] Singh S, Aggarwal BB (1995). Activation of transcription factor NF-κB is suppressed by curcumin (diferuloylmethane) [corrected]. J Biol Chem.

[21] Ali T, Shakir F, Morton J (2012). Curcumin and inflammatory bowel disease: biological mechanisms and clinical implication. Digestion.

[22] Monteleone I, Marafini I, Dinallo V (2017). Sodium chloride-enriched diet enhanced inflammatory cytokine production and exacerbated experimental colitis in mice. J Crohns Colitis.

[23] Hata K, Andoh A, Shimada M (2002). IL-17 stimulates inflammatory responses via NF-κB and MAP kinase pathways in human colonic myofibroblasts. Am J Physiol Gastrointest Liver Physiol.

[24] Akdis M, Aab A, Altunbulakli C (2016). Interleukins (from IL-1 to IL-38), interferons, transforming growth factor β, and TNF-α: receptors, functions, and roles in diseases. J Allergy Clin Immunol.

[25] Valko M, Leibfritz D, Moncol J, Cronin MT, Mazur M, Telser J (2007). Free radicals and antioxidants in normal physiological functions and human disease. Int J Biochem Cell Biol.

[26] Chiurchiù V, Maccarrone M (2011). Chronic inflammatory disorders and their redox control: from molecular mechanisms to therapeutic opportunities. Antioxid Redox Signal.

[27] O'Neill S, Brault J, Stasia MJ, Knaus UG (2015). Genetic disorders coupled to ROS deficiency. Redox Biol.

[28] Sasaki M, Joh T (2007). Oxidative stress and ischemia-reperfusion injury in gastrointestinal tract and antioxidant, protective agents. J Clin Biochem Nutr.

[29] Ullman TA, Itzkowitz SH (2011). Intestinal inflammation and cancer. Gastroenterology.

[30] Patil RH, Naveen Kumar M, Kiran Kumar KM (2017). Dexamethasone inhibits inflammatory response via down regulation of AP-1 transcription factor in human lung epithelial cells. Gene.

[31] Andresen L, Jørgensen VL, Perner A, Hansen A, Eugen-Olsen J, Rask-Madsen J (2005). Activation of nuclear factor κB in colonic mucosa from patients with collagenous and ulcerative colitis. Gut.

[32] Pasparakis M (2008). IKK/NF-κB signaling in intestinal epithelial cells controls immune homeostasis in the gut. Mucosal Immunol.

[33] Yang T, Cao C, Yang J (2017). miR-200a-5p regulates myocardial necroptosis induced by Se deficiency via targeting RNF11. Redox Biol.

[34] Strober W, Fuss IJ (2011). Proinflammatory cytokines in the pathogenesis of inflammatory bowel diseases. Gastroenterology.

[35] Nishida A, Hidaka K, Kanda T (2016). Increased expression of interleukin-36, a member of the interleukin-1 cytokine family, in inflammatory bowel disease. Inflamm Bowel Dis.

[36] Broom OJ, Widjaya B, Troelsen J, Olsen J, Nielsen OH (2009). Mitogen activated protein kinases: a role in inflammatory bowel disease?. Clin Exp Immunol.

[37] Langhorst J, Wulfert H, Lauche R (2015). Systematic review of complementary and alternative medicine treatments in inflammatory bowel diseases. J Crohns Colitis.

