# Supplementary material for: Astaxanthin, a xanthophyll carotenoid, prevents development of dextran sulphate sodium-induced murine colitis
Source: J Clin Biochem Nutr. 2018 Aug 11;64(1):66–72. doi: 10.3164/jcbn.18-47 (PMC6348411; doi:10.3164/jcbn.18-47)
Supplement: Supplemental Table 1 [file jcbn18-47st01.pdf]

Supplemental Table 1. Antibodies used in this study

Antibodies	Clone	Manufacturer
rabbit anti-NFκB p65	C-20	Santa Cruz Biotechnology, Dallas, TX
mouse anti-p-c-Jun	KM-1	Santa Cruz Biotechnology, Dallas, TX
HRP-labeled anti-rabbit IgG		GE Healthcare UK Ltd, Chalfont, UK
mouse anti-Lamin A/C	4C11	Cell Signaling Technology, Beverly, MA
mouse anti-ERK		Cell Signaling Technology, Beverly, MA
mouse anti-p-ERK		Cell Signaling Technology, Beverly, MA
mouse anti-p38		Cell Signaling Technology, Beverly, MA
mouse anti-p-p38		Cell Signaling Technology, Beverly, MA
mouse anti-JNK		Cell Signaling Technology, Beverly, MA
mouse anti-p-JNK		Cell Signaling Technology, Beverly, MA
HRP-labeled anti-mouse IgG		GE Healthcare UK Ltd, Chalfont, UK
rabbit anti-p-c-Jun	D47G9	Cell Signaling Technology, Beverly, MA
mouse anti-NFκB p65	12H11	EMD Millipore Corporation, Billerica, MA
HRP-labeled anti-mouse IgG		Vector Laboratories, Burlingame, CA
