# Supplementary material for: Astaxanthin, a xanthophyll carotenoid, prevents development of dextran sulphate sodium-induced murine colitis
Source: J Clin Biochem Nutr. 2018 Aug 11;64(1):66–72. doi: 10.3164/jcbn.18-47 (PMC6348411; doi:10.3164/jcbn.18-47)
Supplement: Supplemental Table 2 [file jcbn18-47st02.pdf]

Supplemental Table 2. PCR primers used in this study

Gene	Sequence 5'-3'	
IL-1β	sense	CAGGATGAGGACATGAGCACC
	anti-sense	CTCTGCAGACTCAAACTCCAC
IL-6	sense	GACAAAGCCACACTCCTTCAGAGA
	anti-sense	CTAGGTTTGCCGATAGATCTC
TNF-α	sense	ATGAGCACAGAAAGCATGATC
	anti-sense	TACAGGCTTGTCACTCGAATT
IL-36α	sense	GGAAGGACCGTATGTCTCCA
	anti-sense	TGGGTTCTTCAGGACCAGAC
IL-36γ	sense	GTCTATCAATCAATGTGTAAACC
	anti-sense	ATCTTCTGCTCTTTTAGCTGCAAT
β-actin	sense	GTGGGCCGCCCTAGGCACCA
	anti-sense	CGGTTGGCCTTAGGGTTCAGGGGGG
